# Development of a Resonant Microwave Sensor for Sediment Density Characterization

**DOI:** 10.3390/s20041058

**Published:** 2020-02-15

**Authors:** R. Mansour, S. Rioual, B. Lescop, P. Talbot, M. Abboud, W. Farah, G. Tanné

**Affiliations:** 1 IFREMER, REM-GM-LAD, 29280 Plouzane, France; rassoul.mansour@ifremer.fr; 2 Univ Brest, Lab-STICC, CNRS, UMR 6285, F-29200 Brest, France; benoit.lescop@univ-brest.fr (B.L.); philippe.talbot@univ-brest.fr (P.T.); Gerard.Tanne@univ-brest.fr (G.T.); 3 UEGP, Faculté des Sciences, Université Saint Joseph, BP 17-5208 Mar Mikhael, Beyrouth 1104 2020, Liban; maher.abboud@usj.edu.lb (M.A.); wehbeh.farah@usj.edu.lb (W.F.)

**Keywords:** resonant sensor, water content, dielectric characterization, microwave, antenna, sediments

## Abstract

In this study, a sensor based on the development of a planar antenna immersed in sediments dedicated to water content monitoring in this type of material is proposed and experimentally validated. It is produced by a conventional Printed Circuit Board (PCB) manufacturing process on a double-sided metalized FR4 substrate. The sensitivity of the sensor is ensured by the variation of the real part of the complex dielectric permittivity of sediments with water content at around 1 GHz. As shown, in this frequency range, electrode polarization and Maxwell–Wagner polarization effects become negligible, leading to only a bulk water polarization sensitivity. The sensor operates in the reflection mode by monitoring the variation of the resonant frequency as a function of the sediment density through the S_11_ reflection measurements. An experimental sensitivity of $820\;\mathrm{MHz}.g^{-1}.{\mathrm{cm}}^{3}$ was achieved. Despite the simplification of data interpretation at the considered frequency, the influence of ionic species such as NaCl in sediments on the real part of the relative complex dielectric permittivity is highlighted. This demonstrates the importance of considering a second parameter such as the S_11_ level at low frequency or the electrical conductivity to extract the density from the frequency measurements.

## 1. Introduction

The study of sedimentary systems and assessment of geological hazards associated with different external mechanisms (earthquake, tsunami, etc.) requires a characterization of the physical and mechanical properties of the medium such as electrical conductivity and density. Different methods exist for this purpose [1]. The most generalized one is coring followed by laboratory gravimetric analysis. Densities may be also determined by nuclear/X-ray devices due to the fact that sediments absorb more nuclear radiation as the bulk density increases [2,3,4]. Acoustic profilers sensitive to the variation of the acoustic impedance have also been used for decades to measure in-situ properties of subsurface sediments [5,6]. Optical backscattered sensors (OBS) are also very popular for this purpose and measure the turbidity of water and then suspended sediment concentrations [7]. Electromagnetic sensors are based on the variation of dielectric property of materials, i.e., the relative complex dielectric permittivity ε_r_ = ε′ − jε′′. Indeed, the latter is strongly correlated to the water volume content due to the high value of the real part of the relative dielectric permittivity (ε′) of water with respect to other species, about 5 for minerals and 80 for water. However, below 50 MHz, electrode polarization and Maxwell–Wagner polarization affect the dielectric permittivity measurements, leading to erroneous high ε’ values [8,9,10]. Extracting the water content by considering simple mixture models is therefore a challenging task. Within this context, there is a real interest in developing electromagnetic sensors around or above 1 GHz, the limit for which the material can be considered as a simple mineral-water mixture. For example, the Hydra Probe (Stevens Water Monitoring System) [11] is based on frequency domain reflectometry (FDR) at 50 MHz and measures the complex dielectric permittivity of soils to indirectly indicate the volumetric water content and electrical conductivity.

Printed planar resonant microwave sensors [12,13,14,15,16] were recently proposed as promising tools for dielectric material characterization due to their possible integration in microwave integrated devices, low-power consumption and low cost. The operating principle of these sensors is based on a resonant structure that is influenced by the variation of the dielectric property of a material localized in its surrounding environment. The resonant frequency, quality factor and signal amplitude of the device are exploited to derive both the real (ε′) and imaginary (ε′′) parts of the relative complex dielectric permittivity. Development of specific planar microwave resonators was reported in the literature for the determination of the relative humidity in air [17,18,19,20,21,22,23] or water content in concrete and organic coatings [24,25,26,27]. The present work aims to develop a planar antenna, which acts as a microwave resonator for the monitoring of water content in sediments. Kaolinite clay is considered as a substitute for real sediments for laboratory tests. The first part of the study aims to demonstrate that the dielectric property of kaolinite from 0.5 to 10 GHz is related to dipolar relaxation of bulk water without any additional polarization effects. This result encourages the development of antennas in this frequency range and the monitoring of the density via the measurement of the resonant frequency. As is detailed in the following sections, a restricted frequency region was selected to provide a resonant sensor with an acceptable quality factor. Among the different existing topologies of antennas, a patch planar antenna was considered due to its small size and thus possible integration in many devices, for example in multi-sensor cones of penetrometers [28]. The second part of the study focuses therefore on the optimization of the patch antenna immersed in kaolinite, a material with high dielectric losses, and on the discussion on its sensitivity toward the density. The presence of ionic species in pore water is known to enhance polarization effects. Its influence on the sensor’s response is therefore also investigated.

## 2. Materials and Methods

Kaolinite powder (SOKA: Société Kaolinière Armoricaine, Saint-Brieuc, France) with a density of 2.6 g/cm^3^ and de-ionized water were mixed with different ratios to simulate real sediments with several densities. Variation of the densities around 1.6 g/cm^3^ were considered, since this value is close to the mean value of the sediment density distribution found in seawater [29]. Their dielectric properties were measured by an open-ended coaxial probe (Keysight Technologies) from 0.2 to 10 GHz by a network analyzer (Agilent E8364A, 45–50 GHz). Prior to analysis a Short-Open-Load (SOL) calibration was made together with the standard protocol which includes the measurements of the S_11_ parameter in air and de-ionized water. The electrical conductivity of the sediments was simultaneously measured by a conductometer (S-470, Mettler Toledo). Following the achieved results, a patch antenna was optimized and designed on a FR4 substrate (ɛ′ = 4.4; tan δ = 0.02) with a thickness of 1.6 mm that was powered with a coaxial feed. This optimization was done by the HFSS software (ANSYS) and by considering a patch antenna immersed in a material associated with ε′ = 35 and tanδ = 0.1, which simulates sediments of 1.6 g/cm^3^ around 1 GHz. Finally, the optimized planar antenna was produced by a standard Printed Circuit Board (PCB) manufacturing process, here photolithography, and immersed in sediments to experimentally validate the method. The reflection parameter S_11_ was then measured by using the same network analyzer and calibration procedure mentioned above. The step in frequency was 3.6 MHz.

## 3. Results and Discussion

### 3.1. Dielectric Characterization of Kaolinite with Different Densities

**Figure 1:**
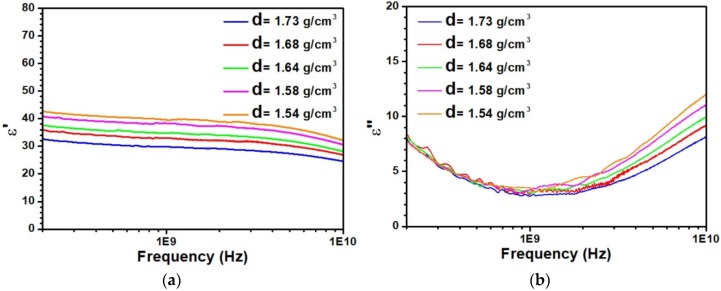
Variation of the relative complex dielectric permittivity as a function of frequency for five different densities (**a**) real part ε’ and (**b**) imaginary part ε ′′.

Figure 1a,b displays the dielectric characterizations of kaolinite with different densities performed by an open-ended coaxial probe from 0.2 to 10 GHz. As observed in Figure 1a, the real part of the relative dielectric permittivity$\;\varepsilon^{\prime }$ is very sensitive to a variation of the density d. Indeed, increasing the water content, i.e., decreasing d, leads to an increase of ε′ in agreement with the high dielectric value of water (about 80) in the considered frequency range. The variation of $\varepsilon^{\prime }\;$with the frequency is relatively weak from 0.2 to 2 GHz. At a higher frequency, the decrease of $\varepsilon^{\prime }\;$is explained by the dipolar relaxation of water molecules. Figure 1b displays the characterization of the imaginary part of the dielectric permittivity $\varepsilon^{\prime \prime }$. A minimum of $\varepsilon^{\prime \prime }$ is clearly highlighted around 1 GHz. At low frequencies, all curves are very close. In contrast, at high frequencies, a clear dependence of $\varepsilon^{\prime \prime }$ as a function of the density d is observed. These experimental findings are very close to the results obtained by Dong and Wang [30]. As detailed by these authors, they are mainly associated with the bulk water polarization. 

**Figure 2:**
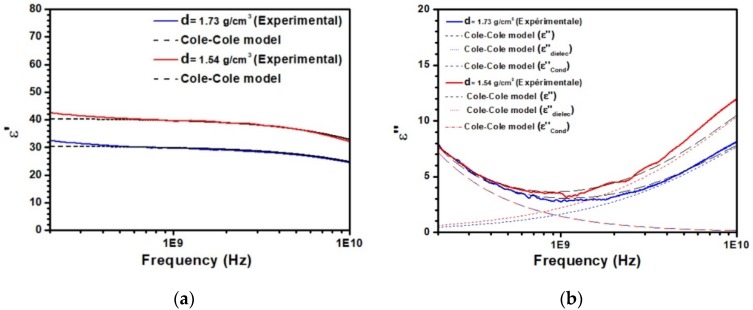
Fitting of the experimental data by the Cole–Cole model for (**a**) the real part and (**b**) the imaginary part of the relative dielectric permittivity. The contributions of the two terms of Equation (2) are displayed in b.

To further discuss these experimental results, the data were compared to the Cole–Cole model [31,32], based on these equations:

(1) $$\varepsilon^{\prime }\;=\;\varepsilon_{\infty }^{\prime }+\frac{\left(\varepsilon_{s}^{\prime }-\varepsilon_{\infty }^{\prime }\right)(1+{\left(\omega \tau \right)}^{1-\alpha }sin\frac{\alpha \pi }{2})}{1+2{\left(\omega \tau \right)}^{1-\alpha }sin\frac{\alpha \pi }{2}+{\left(\omega \tau \right)}^{2\left(1-\alpha \right)}}$$

(2) $$\varepsilon^{\prime \prime }\;=\;\frac{\left(\varepsilon_{s}^{\prime }-\varepsilon_{\infty }^{\prime }\right){\left(\omega \tau \right)}^{1-\alpha }sin\frac{\alpha \pi }{2}}{1+2{\left(\omega \tau \right)}^{1-\alpha }sin\frac{\alpha \pi }{2}+{\left(\omega \tau \right)}^{2\left(1-\alpha \right)}}+\frac{\sigma }{2\pi f\varepsilon_{0}}\;=\;\varepsilon_{dielec}^{\prime \prime }+\varepsilon_{cond}^{\prime \prime }$$

where α is the constant (1≥α>0) related to the distribution of the relaxation time τ, ω is the cyclic frequency of an external electric field change, $\varepsilon_{s}^{\prime }$ and $\varepsilon_{\infty }^{\prime }$ are respectively the static and infinite relative dielectric permittivity constant and σ is the electrical conductivity of the material. Figure 2 displays the comparison between this model and the experimental data for two selected densities. The parameters associated with the Cole–Cole model are given in Table 1. As seen in Figure 2a, the frequency variation of $\varepsilon^{\prime }$ is very well described by the model. At low frequency, below 2 GHz, an almost constant value of $\varepsilon^{\prime }\;$very close to $\varepsilon_{s}^{\prime }$ is observed. At higher frequency, the decrease of ε′ due to dipolar relaxation is also well reproduced. From Table 1, it appears clear that the density depends only on $\varepsilon_{s}^{\prime }$, the two other parameters τ, α remaining constants. Figure 2b focuses on the imaginary part $\varepsilon^{\prime \prime }$ of the relative dielectric permittivity. The contribution of both terms of Equation (2) are represented in this figure. As shown, a good agreement between the model and the data is also achieved. At low frequency, below 0.5 GHz, the second term of Equation (2), which depends on σ, is the main contribution to $\varepsilon^{\prime \prime }$, the first one being negligible. All curves depicted in Figure 1b being nearly identical in this frequency range, fitting the experimental data leads to only one value of the electrical conductivity for the different densities (800 μS/cm). Above 2 GHz, the increase of $\varepsilon^{\prime \prime }$ associated with the relaxation process is well described by the first term of Equation (2). In this case, due to the presence of $\varepsilon_{s}^{\prime }$ in this term, the imaginary part ε’’ also depends on the density. Despite the success of the Cole–Cole model to describe the data correctly, some disagreement on the $\varepsilon^{\prime }$ values at very low frequency are clearly reported. Moreover, the values of the electrical conductivity extracted from the model (σ) with respect to the experimental measurements (σ_exp_) are significantly different. These disagreements observed below 0.5 GHz suggest that some other contribution attributed interfacial polarization or that double layer polarizations may occur at low frequencies. 

The achieved experimental data are of first importance for the development of the sensitive device to the density. Radiofrequency resonators are based on a frequency variation associated with the change of ε′ of the material under monitoring. Here, as observed, a constant value of ε′ independent of the frequency and strongly correlated to the density is found between 0.5 and 2 GHz. Moreover, in between these frequencies, dielectric losses, which impact the quality factor, are minimized. Consequently, this frequency range was selected for the design of the patch antenna. 

### 3.2. Optimization of the Sensitive Patch Antenna

**Figure 3:**
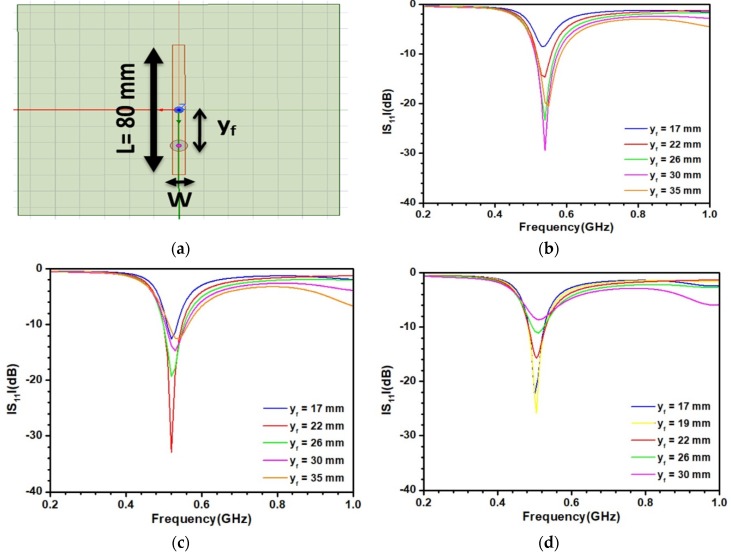
(**a**) Sketch of the antenna and parametric study of the antenna embedded in a material associated with $\varepsilon^{\prime }\;=\;35$, tanδ = 0.1 and operating around 0.5 GHz; (**b**) W = 7 mm, (**c**) W = 5 mm, (**d**) W = 3 mm.

The optimization of the patch antenna was made by considering the antenna powered by a coaxial feed embedded in a material displaying $\varepsilon^{\prime }\;=\;35\;$and tanδ = 0.1, which represents the sediments at about 1 GHz. The sketch of the antenna is depicted in Figure 3a. An antenna length of L = 80 mm was chosen in order to operate around 0.5 GHz, the lowest frequency in the selected frequency range. This value of L is very low with respect to a similar patch antenna designed in air due to the strong influence of $\varepsilon^{\prime }\;=\;35$ on the effective dielectric permittivity. Design of the antenna with values of the width W around 80 mm were first unsuccessfully tested. Only lower values of W provide acceptable results for the optimization of the embedded antenna. Figure 3b–d displays the S_11_ parameter as a function of the localization of the feed y_f_ on the patch with respect to the center of the antenna for widths in the range of a few mm. As observed in Figure 3b, for W = 7 mm, the optimal value of the feed location y_f_ is equal to 30 mm, leading to a value of S_11_ of −30 dBm. For W = 5 mm, as shown in Figure 3c, a similar behavior is found with y_f_ = 22 mm. The location of the feed is then moving toward the center of the patch with respect to the previous case. With a lower value of W, as observed in Figure 3d, a degradation of the reflection parameter is predicted by the antenna. Consequently, the antenna with W = 5 mm and y_f_ = 22 mm was considered to investigate its sensitivity to ε′.

**Figure 4:**
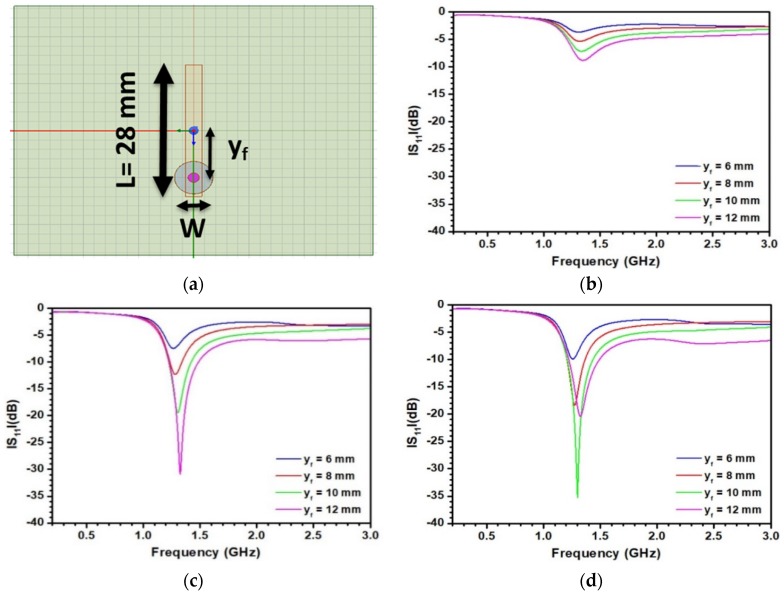
(**a**) Sketch of the antenna and parametric study of the antenna embedded in a material associated with $\varepsilon^{\prime }\;=\;35$, tanδ = 0.1 and operating around 1.3 GHz; (**b**) W = 8 mm, (**c**) W = 4 mm, (**d**) W = 3 mm.

To investigate the influence of the working frequency on the results, a second antenna resonating at a higher frequency was also considered. A sketch of this antenna is depicted in Figure 4a. The antenna’s length is reduced with respect to the first antenna; L is equal to 28 mm to ensure a radiation around 1.3 GHz. Figure 4b–d presents the S_11_ reflection parameter as a function of the width W and localization of the feed y_f_. In Figure 4b, results are displayed for a width W = 8 mm. As observed, increasing y_f_ seems to improve the S_11_ parameter. However, the −10 dB level is never reached, leading to an unacceptable response. Figure 4c presents the reflection parameter for W = 4 mm. In this case, by increasing y_f_, a correct antenna response is achieved. However, such an antenna cannot be fully optimized due to physical limitations of y_f_. Figure 4d displays the reflection parameter S_11_ for a lower value of W (3 mm). In this case, an optimal value of y_f_ (10 mm) associated with a S_11_ value of −35 dB is found. A further decrease of W was not considered due to physical reasons associated with the powering of the antenna by the coaxial port. As the consequence, the configuration depicted in Figure 4d was chosen for testing.

**Figure 5:**
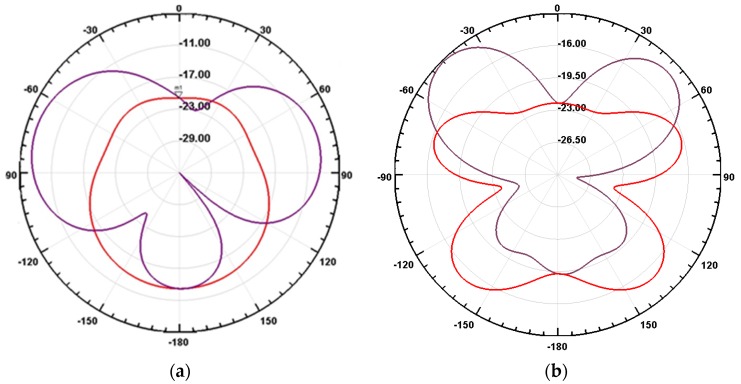
Radiation pattern of antenna operating at 0.5 GHz (**a**) and 1.3 GHz (**b**). (Red pattern: H-plane and purple pattern: E plane).

The designs of the two achieved antennas displayed in Figure 3a and Figure 4a show that the conventional square shape of patch antennas is lost with the optimization procedure associated with the presence of sediments. The term “patch” may therefore lead to erroneous conclusion or misunderstanding and should be suppressed in further applications or discussion, even if patch antennas served as a starting point for the development of such antennas. The radiation patterns of both antenna are presented in Figure 5. As can be seen, the directivity of patch antennas traditionally realized in air is strongly suppressed by the presence of sediments. The maximum gains of the two antennas are indeed very low: −7 dBi and −12.5 dBi at 0.5 and 1.3 GHz, respectively. These values combined together with the high transmission attenuation factor of the material cause transmission experiments to be difficult. As the consequence, the proposed sensor was applied only in a reflection mode by measuring the S_11_ reflection parameter.

### 3.3. Sensitivity of the Patch Antenna to the Density and Experimental Validation

**Figure 6:**
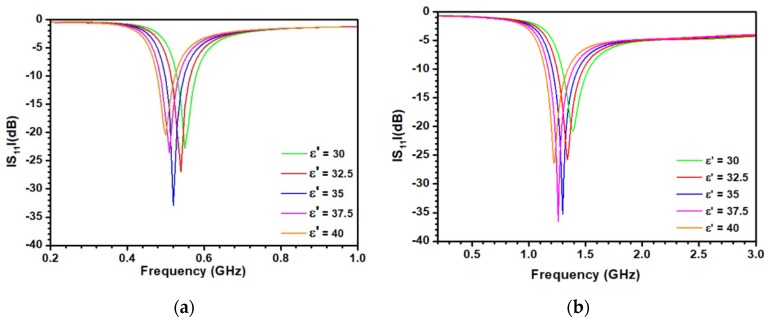

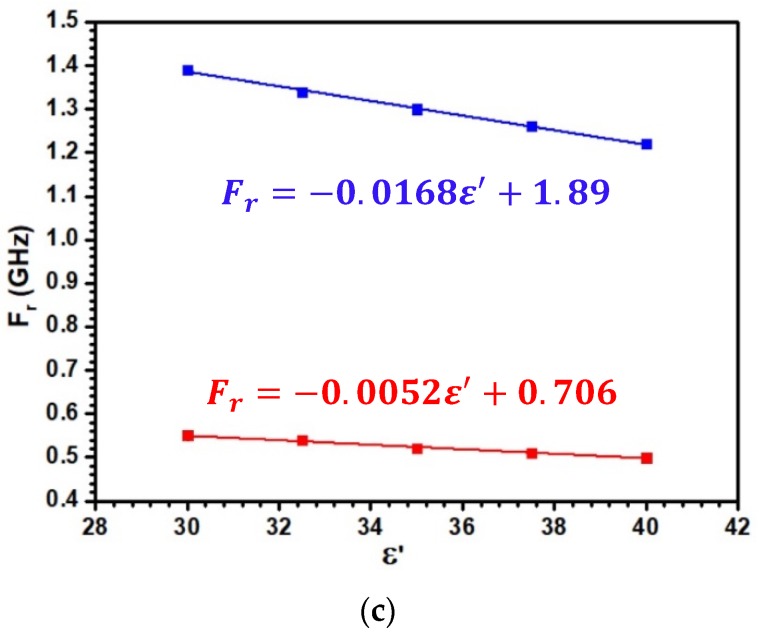
(**a**) Variation of the S_11_ parameter as a function of frequency for different permittivity values. for the antenna operating at 0.5 GHz and (**b**) 1.3 GHz; (**c**) variation of the resonant frequency as a function of the real part of the relative dielectric permittivity for both antennas (red line: antenna working around 0.5 GHz, blue line: antenna working at 1.3 GHz).

Following the optimization stage, the sensitivity of the sensor to the variation of ε′ and hence to d was investigated. For this purpose, electromagnetic simulations were made for the two antennas by covering them by a material displaying several values of $\varepsilon^{\prime }$ while keeping fixed tanδ = 0.1. Results are depicted in Figure 6a for the antenna working around 0.5 GHz. As can be seen, a clear shift of the resonance towards low frequency is associated with an increase of $\varepsilon^{\prime }$, demonstrating thereby the feasibility of the proposed method. We also note a change of the S_11_ level at the working frequency when $\varepsilon^{\prime }$ is varying. This effect is explained by the optimization procedure, which is highly dependent on$\;\varepsilon^{\prime }$. Increasing or decreasing $\varepsilon^{\prime }$ from 35, the value chosen for the optimization, leads to a degradation of the antenna response. Figure 6b displays the results obtained for the second antenna working at 1.3 GHz. As can be seen, a similar behavior is observed. Figure 6c reports the variation of the resonant frequency F_r_ for the two antennas when changing ε′. The frequency variation is linear in the two cases. As detailed in references [33,34], the sensitivity S_ε__′_ of the dielectric resonant method is defined as:

(3) $$S_{\varepsilon^{\prime }}\;=\;\frac{∆F_{r}}{∆\varepsilon^{\prime }}$$

where ∆F_r_ is the shift of the resonance frequency and ∆ε’ the associated variation of the real part of relative dielectric permittivity. In the present case, the sensitivity is constant for a particular antenna and corresponds to the slope of the linear equation depicted in Figure 6b. A higher sensitivity is then obtained at 1.3 GHz. This antenna was therefore selected. 

**Figure 7:**
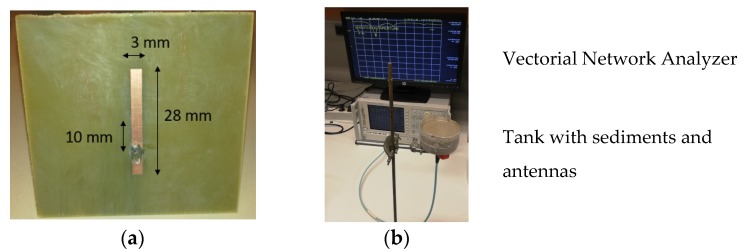
(**a**) Photography of the planar antenna produced on FR4; (**b**) experimental set-up used for the validation of the sensor.

In order to experimentally validate the method, the antenna working at 1.3 GHz was realized by conventional photolithography. Figure 7a presents the antenna on the FR4 substrate (thickness = 1.6 mm). The thickness of the copper sheets is 35 μm. As shown in Figure 7b, the antenna was horizontally placed in a tank and covered by kaolinite. An SMA connector placed on the metallic backplane was used to connect the vectorial network analyzer. The thickness of sediments on the antenna was kept to about 4 cm to ensure no influence of air on the sensor response. Before measurements, the sediments were mixed to get a uniform material and to avoid sedimentation.

**Figure 8:**
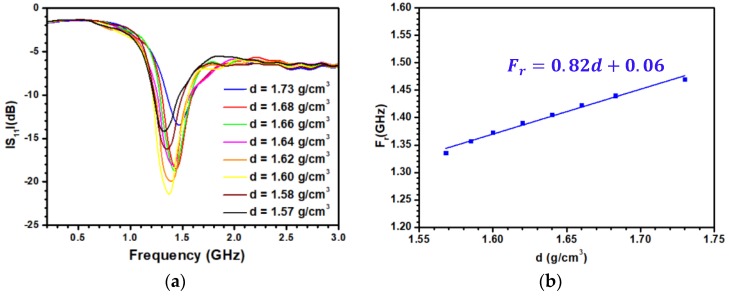
(**a**) Variation of the S_11_ parameter as a function of the frequency for different densities of the sediments for the antenna working at 1.3 GHz; (**b**) variation of the resonant frequency as a function of the density.

Figure 8a displays the change of the experimental S_11_ parameter when the density varies from 1.57 to 1.73 g/cm^3^. The variations of both the resonant frequency and S_11_ value at the resonant frequency can be observed, in agreement with the electromagnetic simulations. The minimum of the S_11_ value achieved for d = 1.6 g/cm^3^ corresponds to the case where the antenna is optimized. Any variation of ε′ or tanδ from this situation leads to an increase of the S_11_ level. Figure 8b presents the variation of the resonant frequency as a function of the density. As shown, a linear fitting provides a very good description of the data in the density range under consideration. The sensitivity S_d_ of the sensor can then be defined as:

(4) $$S_{d}\;=\;\frac{∆F_{r}}{∆\text{d}}\;=\;820\;\mathrm{MHz}\;g^{-1}\;{\text{cm}}^{3}$$

Following the linear fitting displays in Figure 8b, the error on the density ∆d is given by the relation:

(5) ∆d (g/cm^3^) = 1.22 ∆F_r_ (GHz)

where ∆F_r_ is the error on the determination of the resonant frequency. Experimentally, this value is in the order of the frequency step chosen for the S_11_ acquisition. A step of 3.6 MHz leads to ∆d = 4.4 × 10^−3^ g/cm^3^, i.e., a relative uncertainty well below 1%. This value makes the proposed sensor competitive with respect to acoustic sensors with the advantage of limiting the data treatment [35]. Note that the sensor displays such a high resolution due to the choice of the frequency range and to the optimization procedure detailed above. Both enable the retention of a correct resonance shape in the high loss dielectric material. 

**Figure 9:**
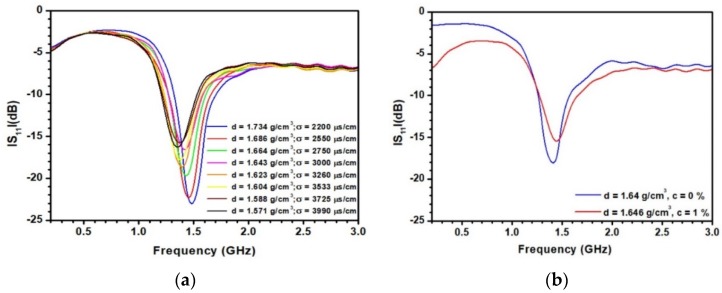
(**a**) Variation of the S_11_ parameter as a function of the frequency for a concentration c = 0.5 %; (**b**) resonances measured at (d = 1.640 g/cm^3^, c = 0 %) and (d = 1.646 g/cm^3^, c = 1 %).

To investigate the influence concentration of ionic species in sediments on the sensor’s response, similar curves to those depicted in Figure 8a were measured at different concentrations c of NaCl (from 0% to 1.5% in mass). As an example, Figure 9a displays the S_11_ parameter as a function of the density for a concentration c = 0.5%. As indicated in the legend of the figure, the variation of the density remains very close to that observed in Figure 8a. However, it is now accompanied by an increase of the electrical conductivity, from 2200 to 4000 μS/cm. This increase of conductivity leads to a decrease of the S_11_ level at low frequency due to the associated radiofrequency losses. A shift of the resonance clearly appears when increasing the density in agreement with the data reported in Figure 8. As discussed above, to investigate a possible effect of polarization electrode initiated by ionic species on the sensor’s response, Figure 9b displays the resonances measured with two conditions: (d = 1.64 g/cm^3^, c = 0%) and (d = 1.646 g/cm^3^, c = 1%). The penetration of ions in sediments leads clearly to a frequency shift associated with a decrease of the ε’. This result is supported by the decrease of the real part of the relative dielectric permittivity of pore water containing NaCl ions with respect to pure water, from 80.5 to 73.5 at c = 3% [36] and was previously exploited to develop a resonant sensor sensitive to water salinity [37] or pH [38]. It confirms also that at the frequency considered here, the material can be considered as a simple mixture of water and mineral with no polarization effects. Indeed, these effects of polarization would lead to the opposite situation with an unrealistic increase of ε’. The two sets of data depicted in Figure 9b being associated with almost the same density of sediments, it is clear that the extraction of the density from presented measurements cannot be made by only considering the measured resonant frequency F_r_. Knowledge of the ionic concentration is required for this purpose. In particular, the S_11_ level at low frequency may be used, since it varies with c.

**Figure 10:**
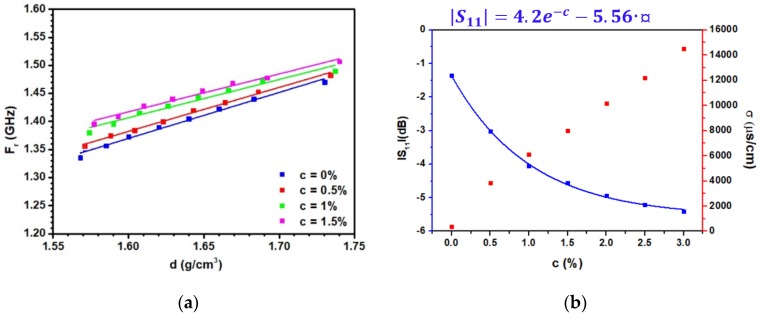
(**a**) The resonant frequency F_r_ as a function of the density for several ionic concentrations c. (**b**) The S_11_ reflection parameter measured at 0.5 GHz and the conductivity σ as a function of the concentration c of NaCl solutions.

Figure 10a presents the resonant frequency as a function of the density for several concentrations. Almost parallel lines can be observed due to the presence of shifts induced by the presence of ionic species in pore water, which reduces the real part of its relative dielectric permittivity. The density d can be extracted from the measured resonant frequency F_r_ via the relation:

(6) $$d\;=\;\frac{F_{r}-\;\alpha \left(c\right)}{\beta \left(c\right)}$$

The values of α and β parameters are reported in Table 2. As can be seen, the β parameter is independent of the concentration, explaining the presence of almost parallel lines in Figure 9a. This is not the case for α (c), which clearly displays a variation with the concentration. The knowledge of the concentration is therefore of crucial importance to extract the density from the measurements. For this purpose, we propose to exploit the results depicted in Figure 9b where the S_11_ level at low frequency is shown to depend on c. Figure 10b presents the S_11_ level measured at 0.5 GHz as a function of c. As can be seen, the S_11_ level is indeed correlated to c and varies with an exponential law. From the measured S_11_ values at 0.5 GHz, the concentration c can therefore be determined, leading to the knowledge of the density d via Equation (6). Fundamentally, the variation of the S11 level at low frequency is induced by the increase of the electrical conductivity. An alternative to the monitoring of c by the S_11_ level is the additional measurement of the electrical conductivity. To provide further insight on this point, the electrical conductivity is also represented in Figure 10b.

## 4. Conclusions

As a summary, the development of planar resonant radiofrequency sensors for water content monitoring in high loss dielectric materials such as sediments is feasible around 1 GHz due to the absence of polarization effects and to the presence of a minimum of the radiofrequency losses. These two points enable (1) the optimization of an antenna in an unfavorable medium and (2) a clear understanding of the observed frequency shift. The realized resonant sensor shows a high resolution with a relative uncertainty of less than 1%, making it competitive with respect to acoustic sensors. In the second part of the study, when dealing with pore water with different ion contents, the feasibility of the proposed method is also proved. In this case, the consideration of only the frequency shift is not sufficient and consideration of either the S_11_ parameter at low frequency or electrical conductivity is mandatory. The proposed resonant sensor, which is compatible with the standard PCB manufacturing process, should therefore considered as a promising innovative method for the monitoring of sediments in geological sciences. 

## Figures and Tables

**Table 1:** Values of the parameters used in the Cole–Cole model detailed in Equations (1) and (2).

d(g/cm^3^)	${\varepsilon^{\prime }}_{s}$	${\varepsilon^{\prime }}_{\infty }$	σ	τ **(s)**	σ (μS/cm)	$\sigma_{\exp}(\mu S/cm)$
1.73	30.6	2	0.2	5 × 10^−12^	800	450
1.68	34	2	0.2	5 × 10^−12^	800	530
1.64	35.6	2	0.2	5 × 10^−12^	800	500
1.58	38.8	2	0.2	5 × 10^−12^	800	510
1.54	40.6	2	0.2	5 × 10^−12^	800	480

**Table 2:** α and β parameters deduced from the fitted curves displayed in Figure 10a.

C (%)	α **(c)**	β **(c)**
0	0.06	0.82
0.5	0.13	0.783
1	0.316	0.682
1.5	0.338	0.674
